# A compact model for magnetic tunnel junction (MTJ) switched by thermally assisted Spin transfer torque (TAS + STT)

**DOI:** 10.1186/1556-276X-6-368

**Published:** 2011-04-28

**Authors:** Weisheng Zhao, Julien Duval, Jacques-Olivier Klein, Claude Chappert

**Affiliations:** 1 IEF, Université Paris-Sud, 15 Rue Georges Clemenceau, Orsay, 91405, France; 2 UMR8622, CNRS, Batiment 220, Campus d'Orsay, 91405, France

## Abstract

Thermally assisted spin transfer torque [TAS + STT] is a new switching approach for magnetic tunnel junction [MTJ] nanopillars that represents the best trade-off between data reliability, power efficiency and density. In this paper, we present a compact model for MTJ switched by this approach, which integrates a number of physical models such as temperature evaluation and STT dynamic switching models. Many experimental parameters are included directly to improve the simulation accuracy. It is programmed in the Verilog-A language and compatible with the standard IC CAD tools, providing an easy parameter configuration interface and allowing high-speed co-simulation of hybrid MTJ/CMOS circuits.

## Background

**Figure 1:**
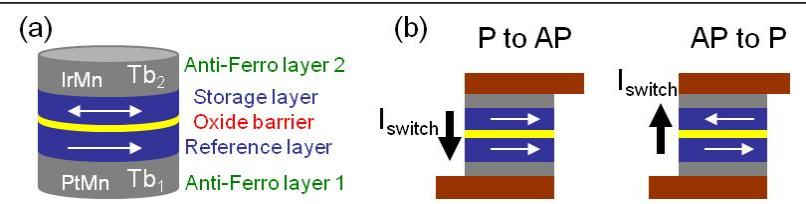
**TAS + STT switching approach**. (a) The MTJ nanopillar with two antiferromagnetic layers. (b) Bidirectional current switches the MTJ between the parallel and anti-parallel state

Spintronics is a very rapidly emerging area of R&D (Nobel Prize 2007) that has the potential to impact significantly on the future of all aspects of electronics beyond CMOS [1]. Magnetic tunnel junctions [MTJ] is one of the most promising spintronic devices for logic and memory applications, which combines magnetism and electronics and promises high write/read speed, non-volatility, infinite endurance etc. [2]. An MTJ is a nanopillar composed of two ferromagnetic [FM] layers and one oxide thin barrier. The Tunnel MagnetoResistance [TMR] phenomenon exists in MTJs [3], which describes the different resistance value R_P _and R_AP _corresponding to the parallel or anti-parallel configuration of the relative magnetization orientations of the two FM layers, respectively. For practical applications, the magnetization direction of one FM layer is pinned as reference and that of the other FM layer is free to store the binary state (see Figure 1a). Recently, TMR = R_AP_-R_P_/R_P _ratio was found to be more than 604% by using the MgO oxide barrier [4] and this allows MTJ to present excellent sensing performance.

Today, most of the R&D efforts in MTJ are focused on its switching approaches, which are expected to be scalable, energy efficient, reliable and fast. A number of approaches have been investigated since 2002, such as thermally assisted switching [TAS] [5] and spin transfer torque [STT] [6,7]. However all of them suffer from either high power or stability issue and cannot meet the requirements for wide applications. Thermally assisted spin transfer torque [TAS + STT] is an emerging approach [8,9], which is based on the temperature dependence of exchange bias storage principle [10], as used in TAS [5]. This switching mechanism involves applying a low current through STT to raise the MTJ temperature above the blocking temperature (*T*_*b*_) of the antiferromagnetic layer associated to the storage layer, resulting in a hysteresis loop centred about zero (see Figure 1). *T*_*b *_depends mainly on the material composition (e.g. ~423K for IrMn and ~573K for PtMn). This method combines the advantages of both TAS and STT technologies, giving the best trade-off among data reliability, power efficiency, speed and density. Unlike other nanodevices [11], MTJ can be easily integrated with CMOS circuits [12]. Based on hybrid MTJ/CMOS [13], innovative memory and logic circuits are expected to provide high performance or new functionalities beyond CMOS. A Spice-compatible efficient compact model for MTJ is an essential requirement for the hybrid MTJ/CMOS design and simulation.

## Physical model integration

This compact model is based on our previous STT MTJ model, which is composed of two sub-modules representing respectively the sensing and switching operations [14]. For sensing, the MTJ resistance and TMR ratio are calculated to obtain respectively *R*_*P *_and *R*_*AP *_[15]. For switching, the STT critical current, *I*_*C*_, calculation model was implemented to obtain the hysteresis loop margin of storage layer [5]. The present model offers an improvement over the previous work [14-17] as it integrates the temperature evaluation and STT dynamic switching models to describe the TAS + STT switching approach. In order to optimise the simulation speed, one of the most important performances for logic and memory designs, some physical phenomena like the oscillating effects during switching are omitted.

### Temperature evaluation model

**Figure 2:**
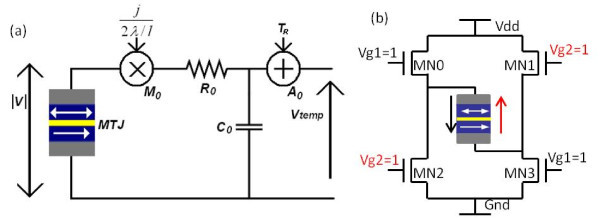
**Circuit implementation for modelling and simulation**. (a) Equivalent electrical RC circuit of temperature evaluation model. (b) MTJ switching circuit; either "Vg1" or "Vg2" is set to '1' to generate the current *I*_*switch*_. RC, resistor/capacitor

The temperature evaluation of MTJ depends on the form and duration of switching current according to Equations 1 and 2 [17,18]. As square current pulses are often used for logic and memory circuit design and simulation, Equation 1 can be then simplified to Equations 3 and 4 to describe respectively the heating and cooling operations driven by a current pulse. This model allows simulating the thermally assisted mechanism of TAS+STT approach. An equivalent electrical circuit corresponding to Equation 1 has been included to monitor the temperature evaluation of MTJ (see Figure 2a), which is based on a simple resistor/capacitor circuit. By adding a multiplier (*M*_*0*_) and an adder (*A*_*0*_), the temperature *T *can be observed in real time through the voltage node *V*_*temp *_(i.e. 1V = 1K). The values of *R*_*0 *_and *C*_*0 *_are set as constant to obtain τ_*th *_calculated by Equation 2,(1)(2)

where *V *is the voltage across MTJ nanopillar, *λ *is thermal conductivity of the thermal barrier, *C*_*V *_is heat capacity per unit volume, *j *is current density, *T*_*R *_is room temperature, thick_b is the thickness of thermal barrier, thick_s is the total thickness of MTJ nanopillar and *τ*_*th *_is the characteristic heating/cooling time. This leads to(3)(4)

where *D*_*heat *_is the heating current pulse duration, *D*_*cool *_is the cooling duration, and *T*_*heat *_and *T*_*cool *_present respectively temperature of MTJ during heating/cooling operations.

### Spin Transfer Torque (STT) dynamic switching model

STT dynamic switching behaviours are described by Equations 5 and 6, which are crucial to simulate the power and speed performances of hybrid MTJ/CMOS circuits. Thermal fluctuations induce an initial angle Θ_0 _between the magnetization of the storage layer and its easy axis [19], which is approximated by Equation 6. High temperature increases Θ_0 _and then reduces the STT switching duration, *D*_*switch*_. STT state reversal depends on switching current value, *I*_*switch*_, which should be higher than the critical current, *I*_*C*_. *D*_*switch *_can be linearly reduced down to according to nanosecond range with high *I*_*switch *_[20]. This property is useful for the design and simulation of hybrid MTJ/CMOS circuits dedicated to logic applications, which require very high speed (e.g. approximately gigahertz).(5)(6)

where *H*_*ani *_is in-plane uniaxial magnetic anisotropy field, *μ*_*0*_*M*_*s *_is saturation field in the storage layer, *α *is Gilbert damping coefficient, *γ*_*0 *_is the gyromagnetic constant, *Vol *is the volume of storage layer and *k*_*B *_is the Boltzmann constant.

## Compact model simulation and validation

### Co-simulation of Hybrid MTJ/CMOS circuit

**Figure 3:**
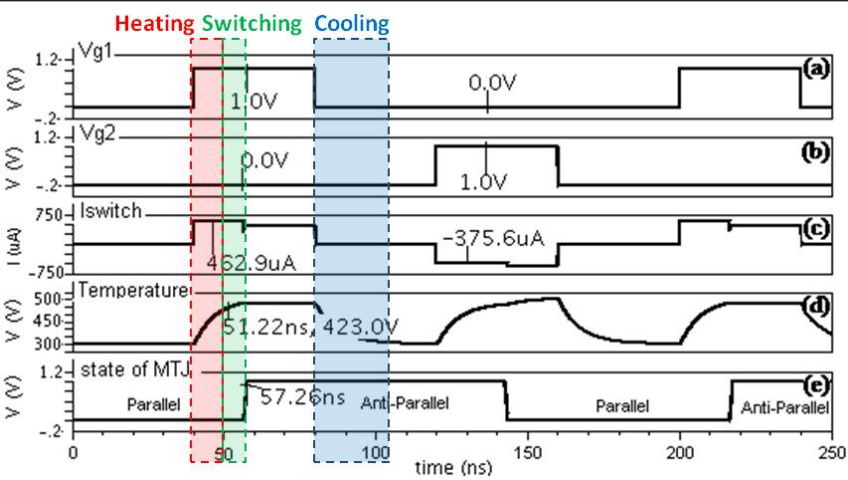
**Transient simulation of compact model**. (a) and (b) Control signals activate the circuit to generate bidirectional currents. (c) MTJ is switched between the P and AP. (d) Temperature evaluation. (e) The state of MTJ. P, parallel; AP, anti-parallel.

This compact model has been developed in Verilog-A language and implemented on Cadence Virtuoso CAD platform [21]. Its default parameters correspond to a MTJ nanopillar BiFe(10)/IrMn(6)/CoFeB(1)/MgO(0.85)/CoFeB(3)/PtMn(6). Thanks to the graphical parameter configuration of Verilog-A, MTJ can be set easily with different material and process parameters. By using CMOS 65 nm design-kit, a simple hybrid circuit (see Figure 2b) [22] has been successfully simulated (see Figure 3), which validates the functionalities and behaviours of this model. The voltage pulse "Vg1" is activated at 40 ns and I_switch _begins to heat the MTJ from ambient temperature. As its temperature reaches up to T_b2 _after ~11.22 ns, the model compares the *I*_*switch *_(approx 462.9 uA) with the STT critical current I_C _(~150 uA) and switches the state of MTJ from parallel [P] to anti-parallel [AP] state in about 6 ns according to the STT dynamic model. As "Vg1" is deactivated, MTJ begins to cool down to ambient temperature. The state can be reversed from AP to P by activating the control signal "Vg2", which generates *I*_*switc*__h _(approx-375.6uA). The *I*_*switch *_values are asymmetric as a constant voltage supply is used in the simulation (e.g. 1V) and the resistance of MTJ changes between two states (*R*_*P *_and *R*_*AP*_). It is important to note that the voltage pulse width should be longer than *D*_*heat*_*+D*_*switch *_to ensure the reliable switching operation [5].

### Power and die area estimation

The silicon area of this hybrid circuit is ~9.8 um^2 ^as the width of NMOS transistors is set to 1 μm to provide *I*_*switch *_much higher than *I*_*C *_and reduce the duration down to some nanoseconds. The whole switching operation of TAS + STT between the P and AP states dissipates ~2.7pJ of energy.

## Conclusions

In this paper, we present the first compact model for MTJ nanopillar switching using the TAS+STT approach. Transient simulations of a hybrid MTJ/CMOS circuit validate its functionalities and demonstrate that it can be useful to calculate the critical circuit performances like speed, power and die area. The easy parameter interface of the Verilog-A language allows us to analyse the characteristics of MTJ with different materials, area and thin film thickness etc. By using this model, a number of hybrid MTJ/CMOS complex circuits are under investigation in our laboratory.

## Competing interests

The authors declare that they have no competing interests.

## Authors' contributions

ZWS, KJO and CC designed the modelling. DJ programmed the model. ZWS and DJ performed the simulation and wrote the manuscript.
